# Supplementary material for: HIVprotI: an integrated web based platform for prediction and design of HIV proteins inhibitors
Source: J Cheminform. 2018 Mar 9;10:12. doi: 10.1186/s13321-018-0266-y (PMC5845081; doi:10.1186/s13321-018-0266-y)
Supplement: Supplementary file 2 — Additional file 2. Source code of HIVProtI web server. [file 13321_2018_266_MOESM2_ESM.zip › HIVprotI_Source-code/hivprotI/weka-3-6-6/doc/weka/classifiers/meta/CostSensitiveClassifier.html]

CostSensitiveClassifier


---


|  |  |  |
| --- | --- | --- |
| **Overview** \| **Package** \| **Class** \| **Tree** \| **Deprecated** \| **Index** \| **Help** \| **Weka's home** | |  |
| **PREV CLASS**   **NEXT CLASS** | **FRAMES**    **NO FRAMES**     **All Classes** |  |
| SUMMARY: NESTED \| FIELD \| CONSTR \| METHOD | DETAIL: FIELD \| CONSTR \| METHOD |  |


---


## weka.classifiers.meta Class CostSensitiveClassifier

```
java.lang.Object
  weka.classifiers.Classifier
      weka.classifiers.SingleClassifierEnhancer
          weka.classifiers.RandomizableSingleClassifierEnhancer
              weka.classifiers.meta.CostSensitiveClassifier
```

**All Implemented Interfaces:**: java.io.Serializable, java.lang.Cloneable, CapabilitiesHandler, Drawable, OptionHandler, Randomizable, RevisionHandler

---

``` public class CostSensitiveClassifier extends RandomizableSingleClassifierEnhancer implements OptionHandler, Drawable ```

A metaclassifier that makes its base classifier cost-sensitive. Two methods can be used to introduce cost-sensitivity: reweighting training instances according to the total cost assigned to each class; or predicting the class with minimum expected misclassification cost (rather than the most likely class). Performance can often be improved by using a Bagged classifier to improve the probability estimates of the base classifier.


Valid options are:

```
 -M
  Minimize expected misclassification cost. Default is to
  reweight training instances according to costs per class
```

```
 -C <cost file name>
  File name of a cost matrix to use. If this is not supplied,
  a cost matrix will be loaded on demand. The name of the
  on-demand file is the relation name of the training data
  plus ".cost", and the path to the on-demand file is
  specified with the -N option.
```

```
 -N <directory>
  Name of a directory to search for cost files when loading
  costs on demand (default current directory).
```

```
 -cost-matrix <matrix>
  The cost matrix in Matlab single line format.
```

```
 -S <num>
  Random number seed.
  (default 1)
```

```
 -D
  If set, classifier is run in debug mode and
  may output additional info to the console
```

```
 -W
  Full name of base classifier.
  (default: weka.classifiers.rules.ZeroR)
```

```
 Options specific to classifier weka.classifiers.rules.ZeroR:
```

```
 -D
  If set, classifier is run in debug mode and
  may output additional info to the console
```

Options after -- are passed to the designated classifier.

**Version:**
:   $Revision: 1.29 $

**Author:**
:   Len Trigg (len@reeltwo.com)

**See Also:**: Serialized Form

---

| **Field Summary** | |
| --- | --- |
| `static int` | `MATRIX_ON_DEMAND`             load cost matrix on demand |
| `static int` | `MATRIX_SUPPLIED`             use explicit cost matrix |
| `static Tag[]` | `TAGS_MATRIX_SOURCE`             Specify possible sources of the cost matrix |

**Fields inherited from interface weka.core.Drawable**

`BayesNet, Newick, NOT_DRAWABLE, TREE`


**Constructor Summary**

`CostSensitiveClassifier()`
  
          Default constructor.


| **Method Summary** | |
| --- | --- |
| `void` | `buildClassifier(Instances data)`             Builds the model of the base learner. |
| `java.lang.String` | `costMatrixSourceTipText()` |
| `java.lang.String` | `costMatrixTipText()` |
| `double[]` | `distributionForInstance(Instance instance)`             Returns class probabilities. |
| `Capabilities` | `getCapabilities()`             Returns default capabilities of the classifier. |
| `CostMatrix` | `getCostMatrix()`             Gets the misclassification cost matrix. |
| `SelectedTag` | `getCostMatrixSource()`             Gets the source location method of the cost matrix. |
| `boolean` | `getMinimizeExpectedCost()`             Gets the value of MinimizeExpectedCost. |
| `java.io.File` | `getOnDemandDirectory()`             Returns the directory that will be searched for cost files when loading on demand. |
| `java.lang.String[]` | `getOptions()`             Gets the current settings of the Classifier. |
| `java.lang.String` | `getRevision()`             Returns the revision string. |
| `java.lang.String` | `globalInfo()` |
| `java.lang.String` | `graph()`             Returns graph describing the classifier (if possible). |
| `int` | `graphType()`             Returns the type of graph this classifier represents. |
| `java.util.Enumeration` | `listOptions()`             Returns an enumeration describing the available options. |
| `static void` | `main(java.lang.String[] argv)`             Main method for testing this class. |
| `java.lang.String` | `minimizeExpectedCostTipText()` |
| `java.lang.String` | `onDemandDirectoryTipText()` |
| `void` | `setCostMatrix(CostMatrix newCostMatrix)`             Sets the misclassification cost matrix. |
| `void` | `setCostMatrixSource(SelectedTag newMethod)`             Sets the source location of the cost matrix. |
| `void` | `setMinimizeExpectedCost(boolean newMinimizeExpectedCost)`             Set the value of MinimizeExpectedCost. |
| `void` | `setOnDemandDirectory(java.io.File newDir)`             Sets the directory that will be searched for cost files when loading on demand. |
| `void` | `setOptions(java.lang.String[] options)`             Parses a given list of options. |
| `java.lang.String` | `toString()`             Output a representation of this classifier |

**Methods inherited from class weka.classifiers.RandomizableSingleClassifierEnhancer**

`getSeed, seedTipText, setSeed`

**Methods inherited from class weka.classifiers.SingleClassifierEnhancer**

`classifierTipText, getClassifier, setClassifier`

**Methods inherited from class weka.classifiers.Classifier**

`classifyInstance, debugTipText, forName, getDebug, makeCopies, makeCopy, setDebug`

**Methods inherited from class java.lang.Object**

`equals, getClass, hashCode, notify, notifyAll, wait, wait, wait`

**Field Detail**

### MATRIX\_ON\_DEMAND

```
public static final int MATRIX_ON_DEMAND
```

:   load cost matrix on demand

    **See Also:**: Constant Field Values

---


### MATRIX\_SUPPLIED

```
public static final int MATRIX_SUPPLIED
```

:   use explicit cost matrix

    **See Also:**: Constant Field Values

---


### TAGS\_MATRIX\_SOURCE

```
public static final Tag[] TAGS_MATRIX_SOURCE
```

:   Specify possible sources of the cost matrix


**Constructor Detail**

### CostSensitiveClassifier

```
public CostSensitiveClassifier()
```

:   Default constructor.


**Method Detail**

### listOptions

```
public java.util.Enumeration listOptions()
```

:   Returns an enumeration describing the available options.

    :   **Specified by:**: `listOptions` in interface `OptionHandler` **Overrides:**: `listOptions` in class `RandomizableSingleClassifierEnhancer`
    :   **Returns:**: an enumeration of all the available options.

---


### setOptions

```
public void setOptions(java.lang.String[] options)
                throws java.lang.Exception
```

:   Parses a given list of options.


    Valid options are:

    ```
     -M
      Minimize expected misclassification cost. Default is to
      reweight training instances according to costs per class
    ```

    ```
     -C <cost file name>
      File name of a cost matrix to use. If this is not supplied,
      a cost matrix will be loaded on demand. The name of the
      on-demand file is the relation name of the training data
      plus ".cost", and the path to the on-demand file is
      specified with the -N option.
    ```

    ```
     -N <directory>
      Name of a directory to search for cost files when loading
      costs on demand (default current directory).
    ```

    ```
     -cost-matrix <matrix>
      The cost matrix in Matlab single line format.
    ```

    ```
     -S <num>
      Random number seed.
      (default 1)
    ```

    ```
     -D
      If set, classifier is run in debug mode and
      may output additional info to the console
    ```

    ```
     -W
      Full name of base classifier.
      (default: weka.classifiers.rules.ZeroR)
    ```

    ```
     Options specific to classifier weka.classifiers.rules.ZeroR:
    ```

    ```
     -D
      If set, classifier is run in debug mode and
      may output additional info to the console
    ```

    Options after -- are passed to the designated classifier.

    :   **Specified by:**: `setOptions` in interface `OptionHandler` **Overrides:**: `setOptions` in class `RandomizableSingleClassifierEnhancer`
    :   **Parameters:**: `options` - the list of options as an array of strings **Throws:**: `java.lang.Exception` - if an option is not supported

---


### getOptions

```
public java.lang.String[] getOptions()
```

:   Gets the current settings of the Classifier.

    :   **Specified by:**: `getOptions` in interface `OptionHandler` **Overrides:**: `getOptions` in class `RandomizableSingleClassifierEnhancer`
    :   **Returns:**: an array of strings suitable for passing to setOptions

---


### globalInfo

```
public java.lang.String globalInfo()
```

:   **Returns:**: a description of the classifier suitable for displaying in the explorer/experimenter gui

---


### costMatrixSourceTipText

```
public java.lang.String costMatrixSourceTipText()
```

:   **Returns:**: tip text for this property suitable for displaying in the explorer/experimenter gui

---


### getCostMatrixSource

```
public SelectedTag getCostMatrixSource()
```

:   Gets the source location method of the cost matrix. Will be one of
    MATRIX\_ON\_DEMAND or MATRIX\_SUPPLIED.

    :   **Returns:**: the cost matrix source.

---


### setCostMatrixSource

```
public void setCostMatrixSource(SelectedTag newMethod)
```

:   Sets the source location of the cost matrix. Values other than
    MATRIX\_ON\_DEMAND or MATRIX\_SUPPLIED will be ignored.

    :   **Parameters:**: `newMethod` - the cost matrix location method.

---


### onDemandDirectoryTipText

```
public java.lang.String onDemandDirectoryTipText()
```

:   **Returns:**: tip text for this property suitable for displaying in the explorer/experimenter gui

---


### getOnDemandDirectory

```
public java.io.File getOnDemandDirectory()
```

:   Returns the directory that will be searched for cost files when
    loading on demand.

    :   **Returns:**: The cost file search directory.

---


### setOnDemandDirectory

```
public void setOnDemandDirectory(java.io.File newDir)
```

:   Sets the directory that will be searched for cost files when
    loading on demand.

    :   **Parameters:**: `newDir` - The cost file search directory.

---


### minimizeExpectedCostTipText

```
public java.lang.String minimizeExpectedCostTipText()
```

:   **Returns:**: tip text for this property suitable for displaying in the explorer/experimenter gui

---


### getMinimizeExpectedCost

```
public boolean getMinimizeExpectedCost()
```

:   Gets the value of MinimizeExpectedCost.

    :   **Returns:**: Value of MinimizeExpectedCost.

---


### setMinimizeExpectedCost

```
public void setMinimizeExpectedCost(boolean newMinimizeExpectedCost)
```

:   Set the value of MinimizeExpectedCost.

    :   **Parameters:**: `newMinimizeExpectedCost` - Value to assign to MinimizeExpectedCost.

---


### costMatrixTipText

```
public java.lang.String costMatrixTipText()
```

:   **Returns:**: tip text for this property suitable for displaying in the explorer/experimenter gui

---


### getCostMatrix

```
public CostMatrix getCostMatrix()
```

:   Gets the misclassification cost matrix.

    :   **Returns:**: the cost matrix

---


### setCostMatrix

```
public void setCostMatrix(CostMatrix newCostMatrix)
```

:   Sets the misclassification cost matrix.

    :   **Parameters:**: `newCostMatrix` - the cost matrix

---


### getCapabilities

```
public Capabilities getCapabilities()
```

:   Returns default capabilities of the classifier.

    :   **Specified by:**: `getCapabilities` in interface `CapabilitiesHandler` **Overrides:**: `getCapabilities` in class `SingleClassifierEnhancer`
    :   **Returns:**: the capabilities of this classifier **See Also:**: `Capabilities`

---


### buildClassifier

```
public void buildClassifier(Instances data)
                     throws java.lang.Exception
```

:   Builds the model of the base learner.

    :   **Specified by:**: `buildClassifier` in class `Classifier`
    :   **Parameters:**: `data` - the training data **Throws:**: `java.lang.Exception` - if the classifier could not be built successfully

---


### distributionForInstance

```
public double[] distributionForInstance(Instance instance)
                                 throws java.lang.Exception
```

:   Returns class probabilities. When minimum expected cost approach is chosen,
    returns probability one for class with the minimum expected misclassification
    cost. Otherwise it returns the probability distribution returned by
    the base classifier.

    :   **Overrides:**: `distributionForInstance` in class `Classifier`
    :   **Parameters:**: `instance` - the instance to be classified **Returns:**: the computed distribution for the given instance **Throws:**: `java.lang.Exception` - if instance could not be classified successfully

---


### graphType

```
public int graphType()
```

:   Returns the type of graph this classifier
    represents.

    :   **Specified by:**: `graphType` in interface `Drawable`
    :   **Returns:**: the type of graph this classifier represents

---


### graph

```
public java.lang.String graph()
                       throws java.lang.Exception
```

:   Returns graph describing the classifier (if possible).

    :   **Specified by:**: `graph` in interface `Drawable`
    :   **Returns:**: the graph of the classifier in dotty format **Throws:**: `java.lang.Exception` - if the classifier cannot be graphed

---


### toString

```
public java.lang.String toString()
```

:   Output a representation of this classifier

    :   **Overrides:**: `toString` in class `java.lang.Object`
    :   **Returns:**: a string representation of the classifier

---


### getRevision

```
public java.lang.String getRevision()
```

:   Returns the revision string.

    :   **Specified by:**: `getRevision` in interface `RevisionHandler` **Overrides:**: `getRevision` in class `Classifier`
    :   **Returns:**: the revision

---


### main

```
public static void main(java.lang.String[] argv)
```

:   Main method for testing this class.

    :   **Parameters:**: `argv` - should contain the following arguments: -t training file [-T test file] [-c class index]


---


|  |  |  |
| --- | --- | --- |
| **Overview** \| **Package** \| **Class** \| **Tree** \| **Deprecated** \| **Index** \| **Help** \| **Weka's home** | |  |
| **PREV CLASS**   **NEXT CLASS** | **FRAMES**    **NO FRAMES**     **All Classes** |  |
| SUMMARY: NESTED \| FIELD \| CONSTR \| METHOD | DETAIL: FIELD \| CONSTR \| METHOD |  |


---
